# Metallic photoluminescence of plasmonic nanoparticles in both weak and strong excitation regimes

**DOI:** 10.1515/nanoph-2023-0884

**Published:** 2024-04-15

**Authors:** Xiaoguo Fang, Jiyong Wang, Min Qiu

**Affiliations:** College of Information Science and Electronic Engineering, Zhejiang University, Hangzhou 310027, China; Key Laboratory of 3D Micro/Nano Fabrication and Characterization of Zhejiang Province, School of Engineering, 557712 Westlake University, 18 Shilongshan Road, Hangzhou 310024, Zhejiang Province, China; Institute of Advanced Technology, Westlake Institute for Advanced Study, 18 Shilongshan Road, Hangzhou 310024, Zhejiang Province, China; Ministry of Education Engineering Research Center of Smart Microsensors and Microsystems, School of Electronics and Information, 12626 Hangzhou Dianzi University, Hangzhou, 310018, China; Westlake Institute for Optoelectronics, Fuyang, Hangzhou 311421, China

**Keywords:** metallic photoluminescence, plasmonic nanoparticles, correlated color temperatures, temperature

## Abstract

The luminescent nature of plasmonic nanoparticles (NPs) has been intensively investigated in recent years. Plasmon-enhanced electronic Raman scattering and the radiation channels of metallic photoluminescence (PL) involving conventional carrier recombinations and emergent particle plasmons are proposed in the past few decades but largely limited to weak excitation regimes. Here, we systematically examine the PL evolution of plasmonic NPs under different excitation power levels. The spectral resonances and chromaticity of PL are investigated within and beyond the scope of geometry conservation. Results indicate the nature of PL in plasmonic NPs could be a process of graybody radiation, including one factor of plasmonic emissivity in the weak excitation regime and the other factor of blackbody radiation in the strong excitation regime. This comprehensive analysis provides a fundamental understanding of the luminescent nature of plasmonic NPs and highlights their potential applications in transient temperature detection at the nanometer scale.

## 1 Introduction

Metallic photoluminescence (PL) has garnered significant attention in recent years due to its wide-ranging applications in imaging [1], medicine [2], and nanolasers [3], [4]. PL was initially observed by Mooradian from bulk copper and gold in 1969, demonstrating its poor efficiency as a luminescent process [5]. In 1986, Boyd et al. found that the lightning-rod effect induced by rough metal surfaces could significantly enhance the absorption process of PL [6]. The emission process, however, followed a classical mechanism picture, which was attributed to the recombination of electron–hole pairs between different energy bands [7], [8], [9]. Dulkeith et al. observed PL from gold nanoparticles (NPs), the spectra of which showed similar profiles as their extinction spectra, proposing a nonradiative relaxation of plasmons and sequentially radiative emission of PL from plasmonic energy levels [10]. Inspired by this work, researchers have devoted considerable efforts to exploring the physical mechanisms of plasmon-mediated PL, benefiting from advancements in nanofabrication technologies [11], [12], [13], [14], [15], [16], [17], [18], [19], [20]. In parallel, researchers also proposed that PL from NPs could be regarded as plasmon-enhanced electronic Raman scattering [21], [22], [23], [24]. In this mechanism, PL emission is considered to be dominated by a coherent inelastic light scattering process. Based on such an assumption, researchers developed various nanothermometers by connecting temperature-dependent distribution of states with the anti-Stoke emissions of NPs [25], [26], [27], [28]. In the end, although several mechanisms have been proposed, the physical origin of NPs PL is still under debate. Besides, most of the current studies on PL of NPs focus on weak excitation conditions, where low excitation laser power is used and potential thermal effects, including thermal damages during the luminescent process, are neglected. The geometrical parameters of NPs and thus their plasmonic nature are largely retained. However, in the strong excitation cases, where the thermal effect of NPs becomes dominant and thermal damages might occur, NPs might lose their plasmonic natures. Consequently, the PL of NPs under strong excitation remains largely unexplored, including their spectral features and underlying physical mechanisms. In this study, we systematically examine the emission spectra of PL under various excitation powers, aiming to shed light on the nature of PL in NPs. We propose that the emission of PL in NPs could be a process of graybody radiation, combining spectral properties observed under both weak and strong excitations. Building upon these physical interpretations, we propose the use of a pyrometer for real-time temperature detection for NPs.

## 2 Results and discussion

**Figure 1:**
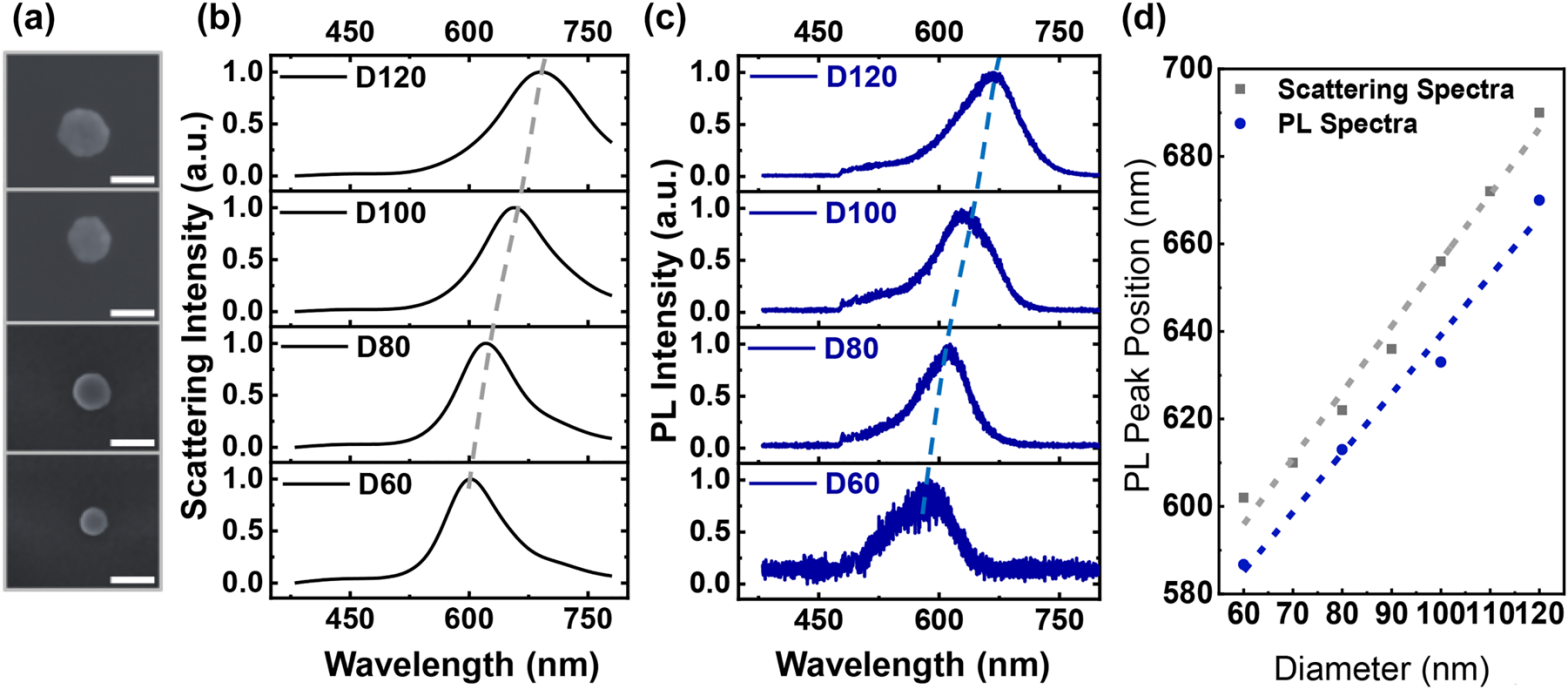
Size-dependent PL of gold NPs in weak excitations. (a) Scanning electron microscope images of gold NPs with the diameter of 60 nm, 80 nm, 100 nm, and 120 nm from the bottom to the top (scale bar: 100 nm). (b) Theoretical scattering spectra of gold NPs with the diameter of 60 nm, 80 nm, 100 nm, and 120 nm from the bottom to the top. The black dashed line indicates the trace of peak position. (c) Experimental PL spectra of gold NPs with the diameter of 60 nm, 80 nm, 100 nm, and 120 nm from the bottom to the top. The blue dashed line indicates the trace of peak position. (d) Comparison of the peak positions between the scattering spectra and PL spectra for gold NPs with different diameters. The black and blue dashed lines indicate the fittings of the peak position for the scattering spectra and the PL spectra, respectively, as functions of the diameter of gold NPs.

Figure 1 shows the PL emission in the weak excitation case. As shown in Figure 1(a), single Au NPs with diameters of 60 nm, 80 nm, 100 nm, and 120 nm are fabricated using standard electron-beam lithography. The inter-distance between two adjacent NPs is 5 μm, so that near-field couplings could be neglected. The total height of gold NPs is 32 nm, including a 2 nm thickness of Cr adhesive layer (see the details from the section “Sample nanofabrications” of Supporting Information). For a simple notation, the NPs with a diameter of m nm are represented by Dm. Figure 1(b) shows corresponding theoretical scattering spectra of gold NPs (see the details from the section “Theoretical scattering spectra of gold NPs” of Supporting Information). As the diameter increases from 60 nm to 120 nm, the peak position of the scattering spectrum, which denotes the plasmonic resonance of in-plane dipolar mode, redshifts from 602 nm to 690 nm. Following the characterization of plasmonic mode, we use a confocal scanning optical microscope (WiTec α300) to measure the PL of single gold NPs (see the details from the section “Optical setups to measure the PL of NPs” of Supporting Information). A continuous-wave laser (wavelength: 473 nm) is used to excite gold NPs. Figure 1(c) shows experimental PL spectra of gold NPs with corresponding diameters. As can be clearly observed, the PL follows a similar profile as scattering spectrum, confirming the radiation channel of particle plasmons (PPs) in the weak excitation case [13], [14], [15], [16], [17]. Figure 1(d) compares peak positions of scattering spectra with PL spectra. They generally follow linear functions against NP diameters and show similar slopes. However, PL spectrum exhibits a generally increasing blue shift as diameter, in comparison with corresponding scattering spectrum. This observation is consistent with reported studies [14], [17].

**Figure 2:**
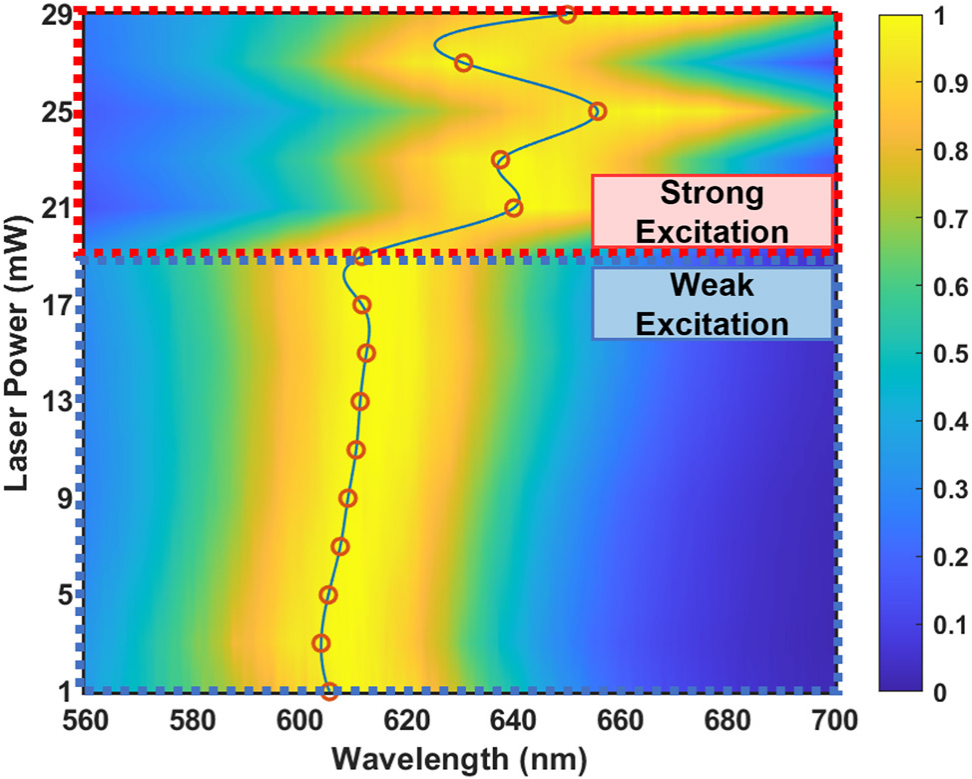
Laser power dependent PL spectra of a gold NP. The diameter of Au NP is 80 nm. Critical laser power at ∼20 mW is observed from PL spectra. The red circles represent the peak positions of PL spectra at the experiments, and the solid blue line is interpolated peak positions of PL spectra.

Figure 2 shows laser power dependent PL spectra for a D80 gold NP. The laser power in the laser spot is gradually increased from 1 mW to 29 mW. A critical laser power at around 20 mW is observed. Below such a critical power, that is the case of weak excitation, PL shows a dominant peak corresponding to the dipolar plasmonic mode. The peak position almost linearly redshifts as the laser power. The full width at half maximum (FWHM) of PL spectrum slightly increases from 69.1 nm to 75.2 nm. When the laser power is beyond the critical value, that is the case of strong excitation, different spectral features of PL can be observed. The central peak position fluctuates in an almost random way, and the linewidth encounters a sudden broadening.

To interpret the inside physical mechanisms, we first consider spectral behaviors of PL with a variance of laser power at weak excitations. In such cases, since the temperature of gold NPs is still below the melting point, their geometrical shapes and thus plasmonic natures are remained. Thus, PL spectra have good reversibility and repeatability. The emission of gold NPs is then dominated by the PPs radiation channel, as seen in the lower panel of Figure 2. Thus, the response of PL in weak excitations to the temperature is similar to that of plasmonic resonance. The temperature dependent plasmonic modes can be attributed to the temperature dependent permittivity. Dispersive permittivity of gold can be predicted using temperature-dependent Lorentz–Drude model [29]:

(1) $$\begin{array}{ll} \varepsilon \left(\omega \right) & =\varepsilon_{\infty }-\frac{\omega_{p}^{2}}{\omega^{2}+i\Gamma_{D}\omega }+\overset{2}{\underset{j=1}{\sum }}C_{j}\Omega_{j}\\ & \;\times \left(\frac{{\text{e}}^{\text{i}\phi_{j}}}{\Omega_{j}-\omega -i{ϒ}_{j}}+\frac{e^{-i\phi_{j}}}{\Omega_{j}+\omega +i{ϒ}_{j}}\right) \end{array}$$

where *ɛ*_∞_ is the high frequency permittivity due to intraband transitions, *ω*_*p*_ is the plasma frequency and Γ_*D*_ is the Drude broadening. *C*_*j*_, Ω_*j*_, ϒ_*j*_ and *ϕ*_*j*_ are the Lorentzian oscillator strength, oscillator energy, oscillator damping and oscillator phase, respectively.

**Figure 3:**
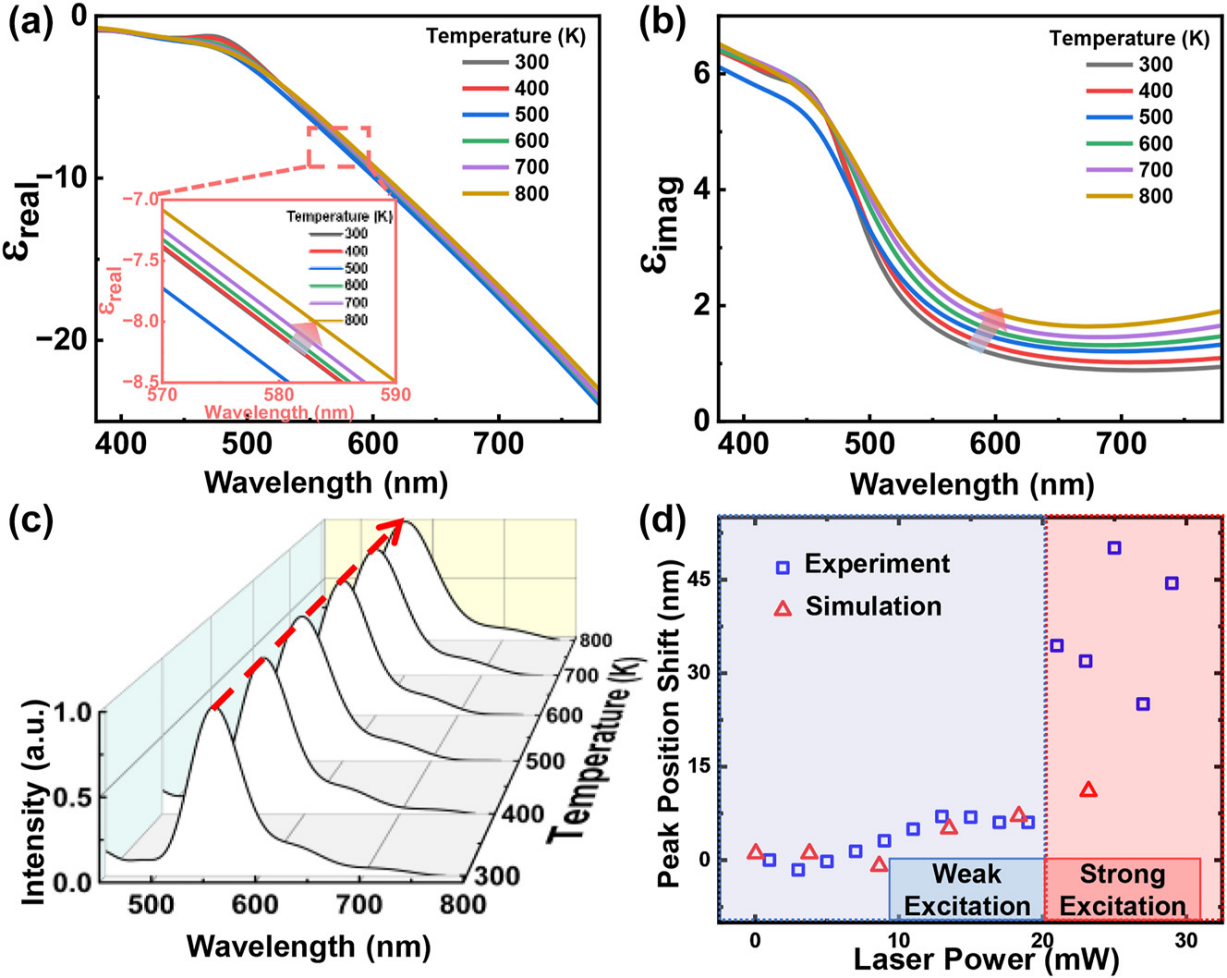
Temperature-dependent PL spectra at weak excitations. The real part (a) and the imaginary part (b) of the dispersive permittivity of gold as functions of temperature. The inset in (a) shows the temperature dependent real part of permittivity in the region of plasmonic resonance. (c) Theoretically temperature-dependent scattering spectra of gold NP with a diameter of 80 nm. (d) Theoretical (triangles) and experimental (squares) peak position shifts against the laser power.

Figure 3(a) and (b) show the dispersive real part and imaginary part of permittivity of gold, respectively, when the temperature increases from 300 K to 800 K. As can be seen, both the real part and the imaginary part increase as the temperature (except 500 K for the real part), which becomes more obvious in longer wavelengths. The increases of real and imaginary parts are responsible for the general redshift of peak position and linewidth broadening of plasmonic resonances, respectively [30]. Figure 3(c) shows theoretical scattering spectra of a gold NP with a diameter of 80 nm if temperature dependent permittivity is taken into account. As can be clearly seen, when the temperature increases from 300 K to 800 K, the peak position of scattering spectrum redshifts from 555.5 nm to 565.8 nm, and the FWHM increases from 62.5 nm to 100.6 nm. We note here that the reshaping of gold NP induced by the high temperature is neglected in theoretical calculations, meaning that plasmonic natures are maintained. However, in reality, the melting point for a gold NP with a diameter around 80 nm is approximatively between 500 K and 795 K [31], [32], [33], beyond which the geometrical conservation of gold NP might be violated. Figure 3(d) compares the peak position shift of theoretical scattering spectra (see the details from “Temperature simulations for gold NPs” of Supporting Information) with that of experimental PL spectra as a function of laser power. When the power is below the critical power ∼20 mW, which is in the regime of weak excitations, it can be observed that scattering spectra and PL spectra show consistent redshifts of peak position as the laser power. When the power is beyond the critical power, that is the case of strong excitations, experimental spectra show distinct tendencies from theoretical predictions. The failure to predict the PL behaviors using the Drude–Lorentz model at strong excitations might stem from two causes: (1) The geometrical conservation is broken at the high temperature, so that intrinsic plasmonic properties are altered (see the details from the section “SEM characterizations of melt gold NPs” of Supporting Information); (2) There might be other radiation channels overweight PPs’ contributions.

**Figure 4:**
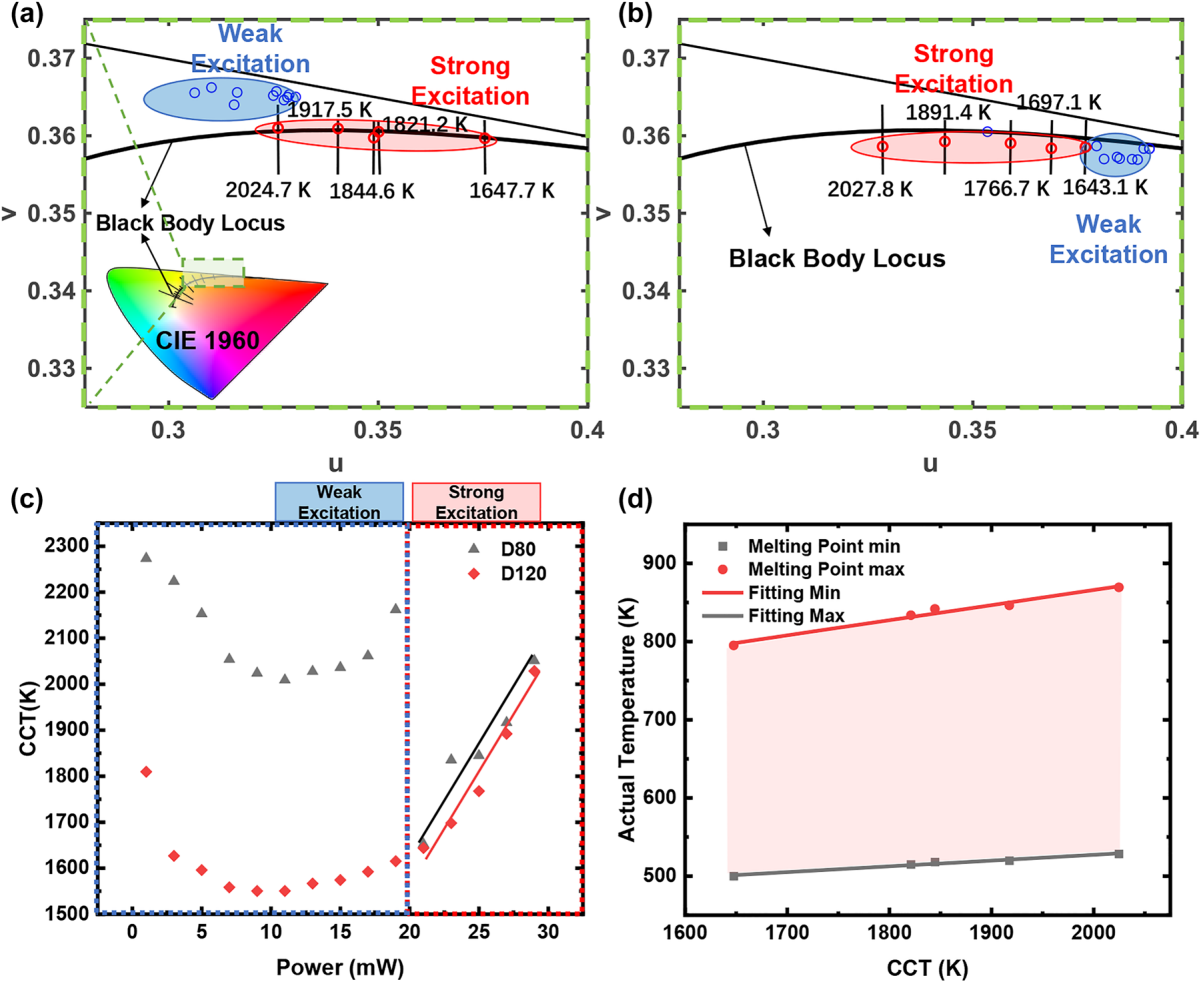
Laser power dependent PL at strong excitations. Chromaticity coordinates and correlated color temperatures (CCTs) of PL spectra of gold NPs with the diameter of 80 nm (a) and 120 nm (b) are characterized in the CIE 1960 color space. Blue areas represent weak excitation cases, and red areas represent strong excitation cases. (c) CCTs of PL spectra of D80 and D120 NPs as functions of laser power; (d) prediction of the actual temperature of gold NPs using CCTs of PL spectra.

When the gold NPs reach strong excitation regimes, as shown in the upper panel of Figure 2, typical features of Lorentzian resonators disappear and are changed into noise-like ones with random peak positions and broadening linewidths, making the identification of PL origin from spectral characteristics difficult. Hence, we introduce a concept compares their spectral integrations in the visible regime, that is color. Figure 4 shows PL in strong excitations characterized by chromatic parameters (see the details from the section “Chromatic parameter calculations” of Supporting Information). As shown in Figure 4(a), chromaticity coordinates of PL spectra under different excitation power for the D80 gold NP are calculated and plotted in the CIE 1960 color diagram. When the gold NP is excited in the strong excitations (red ellipse region), chromaticity coordinates go to the higher CCT region along the blackbody locus as the excitation power. This is quite different from weak excitation cases, in which chromaticity coordinates are almost randomly distributed in a distanced region (blue ellipse) from the blackbody locus. Contrary to almost random spectral properties in terms of peak positions, FWHMs and intensities, the chromatic properties are traceable and reproducible to characterize the PL in strong excitations (see the details from “Reproducibility of chromatic properties of PL in strong excitations” of Supporting Information). Figure 4(b) shows corresponding chromatic characterizations of PL for the D120 gold NP. Similar behaviors can be found, except that chromaticity coordinates of PL in weak excitations locate on the other side of the blackbody locus. Figure 4(c) illustrates the spectral CCTs of PL as functions of excitation powers. In weak excitation conditions, the CCTs of both D80 and D120 initially decrease and subsequently increase as excitation power, showing no consistent tendencies. However, in strong excitation conditions, the CCTs of PL increase nearly linearly from 1647.7 K to 2024.6 K as the laser power. Consequently, the PL of gold NPs in strong excitations could be regarded as a process of blackbody-like radiations. Blackbody-like radiations cast light on potential applications in nanoscale pyrometers by using PL of gold NPs in strong excitations. The actual temperature of gold NPs could be obtained by using the following equation:

(2) $$T_{\text{actual}}=\frac{1}{\frac{1}{T_{\text{CCT}}}+\frac{\lambda }{c_{0}}\ln\left(\sigma \frac{M_{\text{CCT}}}{M_{\text{MPL}}}\right)}$$

where *σ* is a calibration constant, *λ* is the wavelength, and *c*_0_ is a constant (∼1.438 × 10^−2^ m K), *T*_actual_ and *T*_CCT_ represent electronic temperature in thermal equilibrium states. *M*_CCT_ and *M*_MPL_ represent the black body radiation spectrum and the experimental PL spectrum, respectively (see the details from the section “Temperature evaluations using PL of NPs” of Supporting Information). *σ* can be determined through a temperature calibration process. Here, we calibrate *σ* at a critical state where the gold NP starts to melt, since the CCT and theoretical melting point of gold NP in such a state can be found in our experiment and the literature, respectively. The melting point of gold NPs strongly depends on the actual size. For a gold NP with a diameter of around 80 nm, the melting point is within a range (500 K, 795 K) [31], [32], [33]. As shown in Figure 4(d), the actual temperature of gold NPs calculated from Equation (2) is almost a linear function of the CCT, providing a convenient approach to detect the real-time temperature of NPs using PL emission, working like a pyrometer.

In general, combing the spectral properties in both strong and weak excitations, PL could be regarded as a process of graybody radiation:

(3) $$E\left(\lambda ,T\right)=\varepsilon \left(\lambda ,T\right)\cdot W_{\text{bb}}=\varepsilon \left(\lambda ,T\right)\cdot \frac{2\pi hc^{2}}{\lambda^{5}}\frac{1}{\exp\left(\frac{hc}{\lambda k_{B}T}-1\right)}$$

where *h* is the Planck’s constant, *k*_*B*_ is the Boltzmann constant, *c* is the light speed in vacuum, *λ* is the wavelength and *T* is the electronic temperature of NPs. The first factor on the middle of Equation (3) represents the emissivity and the second factor *W*_bb_ represents the blackbody radiation. In an approximation of Kirchhoff’s law, the emissivity is equal to the absorptivity [34], which strongly depends on the geometry of a plasmonic NP. In the weak excitation, as the temperature of NP is low, the second factor could be regarded to be a relatively small constant. The first factor, on the other side, dominates the behavior of PL, exhibiting apparent plasmonic resonance features. However, in strong excitations, as the temperature of NPs becomes sufficiently high and even beyond their melting points, the first factor *ε* (*λ*, *T*) gradually losses the plasmonic nature. In such cases, the second factor *W*_bb_ becomes dominant to reshape PL, giving rise to competitive tendencies in peak position between the redshift due to temperature-dependent permittivity of Au and the blueshift due to temperature-dependent blackbody radiation. By employing formula (3), PL under both weak and strong excitation conditions can be fully described, enabling temperature characterizations of gold NPs through PL in a broad temperature range.

It is worthy discussing about the connections between our concepts and plasmon-enhanced electronic Raman scattering from both the theory and applications aspects. In plasmon-enhanced electronic Raman scattering theory, the PL intensity can be expressed as [28]:

(4) $$E\left(\lambda ,T\right)=\frac{I^{\text{PL}}\left(\lambda ,\lambda_{\text{exc}},T\right)}{I^{\text{exc}}\left(\lambda_{\text{exc}}\right)}=Cf_{\text{PL}}\left(\lambda ,\lambda_{\text{exc}}\right)n\left(\lambda ,\lambda_{\text{exc}},T\right)$$

where *I* ^PL^ and *I* ^exc^ represent the PL emission and excitation intensity, respectively. *T* is the electronic temperature of NPs, and *C* is a constant. *f*_PL_ follows the surface plasmon resonance with a Lorentzian shape [26], [28], *n* is the temperature dependent distribution of states responsible for the anti-Stoke emission, which satisfies a Bose–Einstein distribution in most of literature. Thus,

(5) $$n\left(\lambda ,\lambda_{\text{exc}},T\right)={\left[\exp\left(\frac{E\left(\lambda \right)-E\left(\lambda_{\text{exc}}\right)}{k_{B}T}\right)-1\right]}^{-1}$$

If we compare Equations (3) and (4), it is found that the two mechanisms share similar mathematic laws. From the aspect of physical nature, the two mechanisms might reveal the similar rules of hot electron radiations but from different physical perspectives.

Regarding the application, that is nanoscale pyrometers or nanothermometers [25], [26], [27], the reasons why the chromaticity of PL reflects the temperature of NPs could be understood from two aspects. First, it is natural to link the color with the temperature of metals in our daily routines, for example in iron forge or steel making. That is also the origin for the definition of color temperature. Second, from the standing point of electronic Raman scattering, the chromaticity coordinate w (or z in the CIE 1931 color space) to some extents represents the integrated intensity of PL in high frequencies [35], [36], corresponding to the ratio of anti-Stoke emission to Stoke emission. As the chromaticity coordinates satisfy the constrain *u* + *v* + *w* = 1 in accordance with the CIE 1960 standard, the trace of chromaticity coordinates (*u*, *v*) in the CIE chromatic diagram indeed reflects that ratio and thus the temperature of NPs.

## 3 Conclusions

We systematically investigate the PL evolution of plasmonic NPs with various excitation laser powers. In the weak excitation, the plasmon-like emission channel dominates, tailoring PL spectra to similar profiles as their scattering spectra. With the laser power increases, the plasmon-tailored PL exhibits a consistent redshift in peak position and a broadening in spectral linewidth, which could be attributed to the temperature dependent permittivity of gold. Upon reaching the strong excitation regime, the PL of NPs losses its plasmonic features due to geometry changes. Instead, PL is characterized by using chromatic properties, which imply spectral integrations of PL in blue, green, and red wavelength bands. Remarkably, chromatic coordinates of PL follow the blackbody locus in standard CIE color spaces and the correlated color temperature exhibits a linear function of laser power, indicating the NP in such cases emit the light like a black body. By considering spectral behaviors of PL in both weak and strong excitations, we propose that the PL emission of NPs could be fully described via a process of graybody radiation. This study offers a fundamental perspective on the luminescent nature of plasmonic NPs and provides insights for advanced applications in transient temperature detection at the nanometre scales.

## Supplementary Material

Supplementary Material Details
