# Supplementary material for: Metallic photoluminescence of plasmonic nanoparticles in both weak and strong excitation regimes
Source: Nanophotonics. 2024 Apr 15;13(18):3355–61. doi: 10.1515/nanoph-2023-0884 (PMC11501396; doi:10.1515/nanoph-2023-0884)
Supplement: Supplementary file 1 — Supplementary Material Details [file j_nanoph-2023-0884_suppl_001.docx]

*Supporting Information*

**Metallic photoluminescence of plasmonic nanoparticles in both weak and strong excitation regimes**

Xiaoguo Fang^1,2,3^, Jiyong Wang^4,*^, MIN QIU^2,5,3*^

*1College of Information Science and Electronic Engineering, Zhejiang University, Hangzhou 310027, China.*

*2 Key Laboratory of 3D Micro/Nano Fabrication and Characterization of Zhejiang Province, School of Engineering, Westlake University, 18 Shilongshan Road, Hangzhou 310024, Zhejiang Province, China.*

*3 Institute of Advanced Technology, Westlake Institute for Advanced Study, 18 Shilongshan Road, Hangzhou 310024, Zhejiang Province, China.*

*4 Ministry of Education Engineering Research Center of Smart Microsensors and Microsystems, School of Electronics and Information, Hangzhou Dianzi University, Hangzhou, 310018, China.*

*5 Westlake Institute for Optoelectronics, Fuyang, Hangzhou 311421, China.*

**Corresponding author*: [jiyongwang@hdu.edu.cn](mailto:jiyongwang@hdu.edu.cn); [qiumin@westlake.edu.cn](mailto:qiumin@westlake.edu.cn)

**Sample nanofabrication**

A 100 nm thickness of SiO2 is grown on the cleaned Si substrate by PECVD (SAMCO Inc. PD-220NL). Electron-sensitive resist (All Resist, Arp 6200.09) is spin-coated on the SiO2/Si substrate at a speed of 6000 rpm to attain a thickness ~ 165 nm. After the spin-coating, the sample is baked on a hot plate at 150 ℃ for 1 minute. Electron-beam lithography (EBL) is conducted in RAITH 150 system using an accelerating voltage of 30 kV and a beam current of 150 pA. An optimized dose of 240 μC/cm^2^ is used to obtain high-resolution nanostructures. After electron-beam exposures, the sample is developed with the developer (1:3 MIBK/IPA) at room temperature for 1 minute and then directly dried with stable N_2_ gas flow. A 30 nm gold layer is deposited over a 2 nm Cr adhesion layer by using high vacuum evaporation (ULVAC (Suzhou) Co., Ltd. ei-5z). The working pressure during evaporation is below $5\times{10}^{-6}$ Torr. During the whole evaporation process, the temperature of chamber remains at 20 ℃, and the sample rack rotates at a speed of 50 rpm to ensure the uniformity of deposition. Lift-off is realized by immersing the sample in N-methyl pyrrolidone (NMP) solvent at a temperature of 70 ℃.


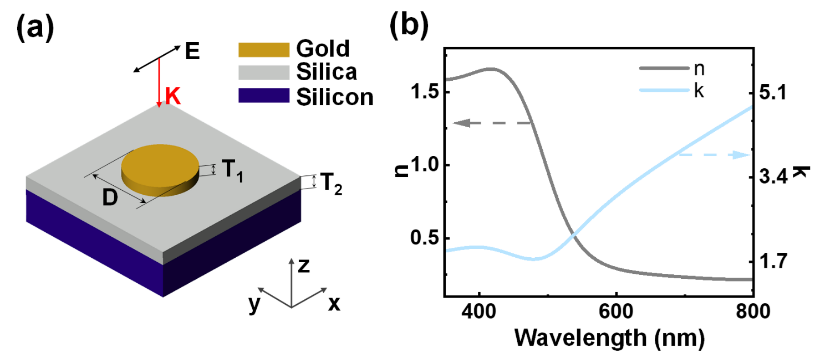
**Theoretical scattering spectra of gold NPs**

**Fig. S1:** Theoretical simulation of the scattering spectrum of a single gold NP. (a) Optical model of a gold NP excited by a linearly polarized and normally incident light. (b) Dispersion relation of a 30 nm gold film measured by an ellipsometer.

In order to calculate scattering spectra of gold NPs, we use the finite-difference time-domain (FDTD) method to model the plasmonic nanostructure. In such a model, as shown in Figure S1(a), the gold NP with the diameter D (range from 80 to 120 nm), and thickness T1 = 30 nm locates on a silica (with a thickness T2 = 100 nm)/silicon substrate. For simplicity, the chromium adhesion layer is not considered in the model. The refraction index of gold is obtained from the ellipsometry measurements as is shown in Figure S1(b). The refraction indices of silica and silicon are 1.46 and 3.88, which are from the database of Palik [1]. The NP is excited by a light with a normal **K** vector, linear polarization along the x-axis, and a wavelength varying from 380 to 780 nm. The model system is enclosed with a perfectly matched layer (PML). The mesh size for the silica/silicon substrate region is 5 nm, and for the gold NP is 2 nm.


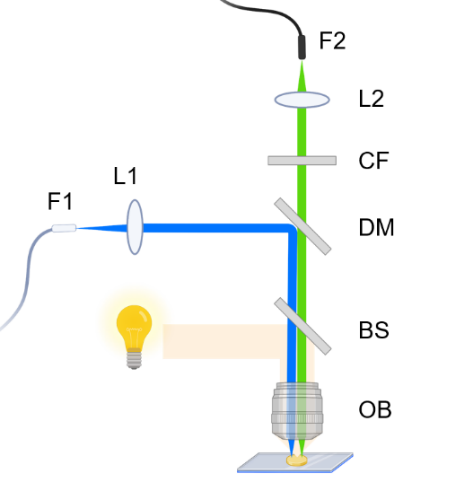
**Optical setups to measure the PL of NPs**

**Fig. S2:** The schematic optical path of the confocal scanning optical microscope. F1 and F2 are optical fibers. L1 and L2 are lenses. CF is a color filter. DM is a dichroic mirror. BS is a beam splitter. OB is objective.

The metallic photoluminescence (PL) spectra of single gold nanoparticles (NPs) are measured using a confocal scanning optical microscope (WiTec α300RAS), as shown in Figure S2. In such an optical microscope, the single gold NP can be observed using the darkfield mode of a high-resolution optical objective (OB, Zeiss Epiplan BD 50X objective lens, NA = 0.55, WD = 9.1 mm).

The core of the optical fiber F2 acts as a pinhole for confocal microscopy. The laser from F1 is sequentially passed through lens (L1), a dichromic mirror (DM), a beam splitter (BS) and OB, and finally focused onto the sample. Before the PL spectra are collected with a CCD spectrometer (WiTec, UHTS 600 SMFC VIS), it is necessary to introduce a color filter (CF) to exclude fundamental scattering and Raman signals. The optical fiber F2 guides the PL into the CCD, acting as a pinhole. The integration time is 60 s and 5 s for weak excitation and strong excitation, respectively. As an example, Figure S3(a) shows the measured PL spectra of a gold NP with the diameter of 80 nm with various laser powers, corresponding to the result of Figure 2 in the main text. Figure S3(b) shows their integrated PL intensity as a function of pump laser power. As can be seen from the Figure S3(b), in the weak excitation (<20 mW), the PL intensity almost shows a linear dependence on the laser power, while it becomes suddenly nonlinear and fluctuate in the strong excitation (>20 mW). The emission efficiency is generally enhanced in the strong excitation case, as thermal radiation (blackbody radiation) is involved. At such a state, the gold NP might be melting and even boiling in a higher pumping power, the thermal radiation part is temporally unstable in the experiment.

**Fig. S3:** MPL spectra of a gold NP with a diameter of 80nm. (a) MPL spectra excited by various excitation powers (b) MPL intensity evolution excited by various laser powers. The experimental data (blue open circles) are fitted by a linear function (red dashed line) in the weak excitation regime.

**
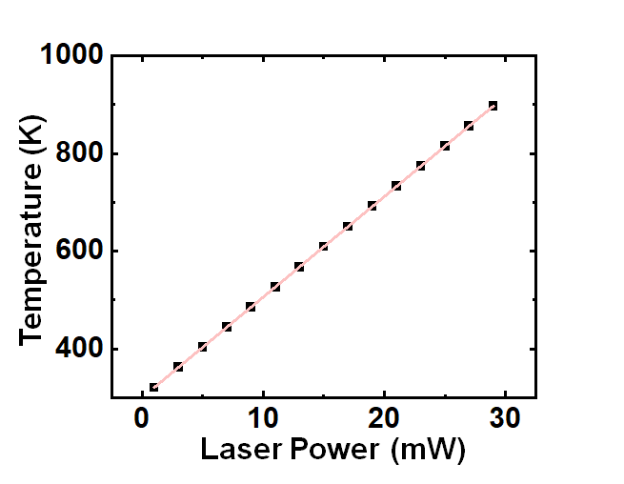
Temperature simulations for gold NPs**

**Fig. S4:** Simulation results of laser-induced heating on gold nanoparticles.

The steady-state temperature simulations for single gold NPs are conducted using the finite element method (COMSOL Multiphysics). The same model system shown in Figure S1(a) is used. The refraction index of gold is obtained from the ellipsometry measurements as shown in Figure S1(b). The imaginary part of the refractive index of silicon is 0.067, which is obtained from the database of Schinke [2]. In such a modelling system, the NP is excited by a Gaussian beam with a waist radius of 600 nm (λ = 473 nm). The heat transfer between solids and convective heat flux between solids and air are calculated using the following equations:

$$\begin{aligned} \nabla\cdot\left( -k\nabla T \right)=Q\#\left( S1 \right) \end{aligned}$$

$$\begin{aligned} q_{0}=h\cdot\left( T_{\text{ext}}-T \right)\#\left( S2 \right) \end{aligned}$$

where *k* is the thermal conductivity, *T* is the solid temperature, *Q* is the heat source, *q_0_* is the heat flux, *h* is the convective heat transfer coefficient (for air [3], h = 10 W/(m2·K)) and Text is the ambient temperature (300 K). The thermal conductivities of gold, silica, and silicon are 317 W/(m·K), 1.4 W/(m·K), and 130 W/(m·K), respectively [4]. Here *Q* is mainly from the optical absorption of gold and silicon, which can be calculated using 1/2*ε_imag_*|*E*|^2^, where *ε_imag_* is the imaginary part of the complex permittivity of gold and silicon, and *E* is the electric field strength. *q_0_* is the heat flux, which is used to calculate the heat dissipation of solids into air. As shown in Figure S4, the simulated temperatures of a single gold NP are approximately a linear function of the laser power.

SEM characterizations of melt gold NPs

**Fig. S5:** SEM images of melt gold NPs. The red dashed circles represent the original shapes and sizes of gold NPs before any thermal damages.

**Chromatic parameter calculations**

In order to calculate the CCTs of PL spectra, three steps for post-processes are necessary to follow:

(1) Extraction and then normalization of PL spectra

The PL spectra can be extracted from the original spectra by excluding the fundamental laser signals and replacing with proper Gaussian interpolations. After that, all the PL spectra are normalized, as shown in Figure 1(c) in the main text.

(2) Calculation of chromaticity coordinates

Firstly, the tristimulus values X, Y, and Z are calculated in according to:

$$\begin{aligned} \left\{ \begin{aligned} X=\sum_{380}^{780} P\left( \lambda\right)\bar{X}\left( \lambda\right)d\lambda\\ Y=\sum_{380}^{780} P\left( \lambda\right)\bar{Y}\left( \lambda\right)d\lambda\\ Z=\sum_{380}^{780} P\left( \lambda\right)\bar{Z}\left( \lambda\right)d\lambda\end{aligned} \right.\#\left( S3 \right) \end{aligned}$$

where $P\left( \lambda\right)$ is the PL spectrum, $\bar{X}\left( \lambda\right)$, $\bar{Y}\left( \lambda\right)$ and $\bar{Z}\left( \lambda\right)$ are color matching functions. The chromaticity coordinates in the CIE 1931 color space can then be calculated as x=X/(X+Y+Z) and y= Y/(X+Y+Z).

The chromaticity coordinates (u, v, w) in the CIE 1960 color space could be obtained by using the following linear transfers [5]：

$$\begin{aligned} \left\{ \begin{aligned} u=\frac{4x}{-2x+12y+3} \\ v=\frac{6y}{-2x+12y+3} \\ w=\frac{-6x+6y+3}{-2x+12y+3} \end{aligned} \right.\#\left( S4 \right) \end{aligned}$$

(3) Calculation of CCTs

The CCT can be either directly indexed from (x, y, CCT) table under the standard of CIE 1931 or calculated from the following formulas [6]:

**Fig. S6:** Power dependent chromatic properties of PL of Au NPs. (a) and (b) show chromaticity coordinate distributions of PL of two Au NPs with the diameter of 80 nm in the CIE 1960 color diagram. The blue circles represent the case of weak excitation, and the red circles represent the case of strong excitation. (c) Power dependent CCTs of PL of the two Au NPs.

$$\begin{aligned} n=\frac{x-0.3320}{0.1858-y}\#\left( S5 \right) \end{aligned}$$

$$\begin{aligned} CCT=437n^{3}+3601n^{2}+5517\#\left( S6 \right) \end{aligned}$$

According to formulas S3 and S4, the PL spectra of D80 NPs under a range of laser powers can be converted to a series of coordinates corresponding to CIE1960 as shown in Fig. S6 (a, b). Through formulas S5 and S6, the relationship between the correlated color temperature and excitation power of the luminescence spectrum of the two particles can be obtained, as shown in Figure S6(c).

**Temperature evaluations using PL of NPs**

The emission spectra of a perfect black body at CCT and a gray body at the actual temperature can be expressed respectively as bellows:

$$\begin{aligned} W_{bb}\left( \lambda,T \right)=\frac{{2\pi hc}^{2}}{\lambda^{5}}\frac{1}{exp \left( \frac{hc}{\lambda k_{B}T}-1 \right)}=\frac{c_{1}}{\lambda^{5}}\frac{1}{e^{\frac{c_{2}}{\lambda T_{CCT}}}-1}\#\left( S7 \right) \end{aligned}$$

$$\begin{aligned} M_{MPL}=\varepsilon W_{bb}\left( \lambda,T_{CCT} \right)=\varepsilon\frac{c_{1}}{\lambda^{5}}\frac{1}{e^{\frac{c_{2}}{\lambda T_{actual}}}-1}\#\left( S8 \right) \end{aligned}$$

where $\varepsilon$ represents emissivity, $\lambda$ is wavelength, $c_{1}=2\pi hc^{2}\approx3.742\times{10}^{-16}W\cdot m^{2}$ and $c_{2}=hc/K_{B}\approx1.438\times{10}^{-2} m\cdot K$. According to Kirchhoff's law, emissivity *ε* is equal to absorptivity *σ*_abs_ at the thermal equilibrium [7]. When the object is a perfect black body, *σ*_abs_ is equal to 1.

The absorptivity *σ*_abs_ of a gray body can be assessed using the following formula:

$$\begin{aligned} \sigma_{abs}\approx\frac{M_{MPL}}{M_{CCT}}\cdot e^{\frac{c_{2}}{\lambda}\left( \frac{1}{T_{\text{actual }}}-\frac{1}{T_{CCT}} \right)}\#\left( S9 \right) \end{aligned}$$

In an inverse process, if the *σ*_abs_ is known, the actual temperature of a gray body excited with a certain laser power can be calculated using the following formula:

$$\begin{aligned} T_{\text{actual }}=\frac{1}{\frac{1}{T_{CCT}}+\frac{\lambda}{c_{2}}\ln\left( \sigma_{abs}\frac{M_{CCT}}{M_{MPL}} \right)}\#\left( S10 \right) \end{aligned}$$

References

[1] E. D. Palik, Handbook of Optical Constants of Solids. Academic press, 1998.

[2] C. Schinke et al., "Uncertainty Analysis for the Coefficient of Band-to-band Absorption of Crystalline Silicon," Aip Advances, vol. 5, no. 6, 2015. https://doi.org/10.1063/1.4923379.

[3] Y. Zhang et al., "Electrically Reconfigurable Non-volatile Metasurface Using Low-loss Optical Phase-change Material," Nature Nanotechnology, vol. 16, no. 6, pp. 661-666, 2021. https://doi.org/10.1038/s41565-021-00881-9.

[4] Stockholm, COMSOL AB, COMSOL User Manual, 5.4 ed., 2012.

[5] J. Schanda, Colorimetry: Understanding the CIE System. John Wiley & Sons, 2007.

[6] C. S. McCamy, "Correlated Color Temperature as an Explicit Function of Chromaticity Coordinates," Color Research & Application, vol. 17, no. 2, pp. 142-144, 1992. https://doi.org/10.1002/col.5080170211.

[7] B. Zhao, C. Guo, C. A. Garcia, P. Narang, and S. Fan, "Axion-field-enabled Nonreciprocal Thermal Radiation in Weyl Semimetals," Nano Lett., vol. 20, no. 3, pp. 1923-1927, 2020. https://doi.org/10.1021/acs.nanolett.9b05179.
